# Effects of Purified *Saccharomyces cerevisiae* (1→3)-β-Glucan on Venous Ulcer Healing

**DOI:** 10.3390/ijms13078142

**Published:** 2012-07-02

**Authors:** Sarah Dantas Viana Medeiros, Sara Lima Cordeiro, Jéssica Escorel Chaves Cavalcanti, Karina Mendes Melchuna, Aleida Maria da Silva Lima, Irami Araújo Filho, Aldo Cunha Medeiros, Keyla Borges Ferreira Rocha, Elizabeth Maia Oliveira, Eduardo Dantas Baptista Faria, Guilherme Lanzi Sassaki, Hugo Alexandre Oliveira Rocha, Valéria Soraya Farias Sales

**Affiliations:** 1 Laboratory of Clinical Immunology, Department of Clinical and Toxicological Analysis, Federal University of Rio Grande do Norte (UFRN), General Gustavo Cordeiro de Farias Ave., Petrópolis, Natal, RN 59012-570, Brazil; E-Mails: sarah_farma@hotmail.com (S.D.V.M.); jessy_ecc@hotmail.com (J.E.C.C.); karinamelchuna@yahoo.com.br (K.M.M.); aleida_maria85@yahoo.com.br (A.M. S.L.); 2 Laboratory of Biotechnology of Natural Polymers (BIOPOL), Department of Biochemistry, Federal University of Rio Grande do Norte (UFRN), 3000, Salgado Filho Ave., Natal, RN 59078-970, Brazil; E-Mails: sara-cordeiro@hotmail.com (S.L.C.); hugo@cb.ufrn.br (H.A.O.R.); 3 University Hospital Onofre Lopes, Department of Surgery, Federal University of Rio Grande do Norte (UFRN), 620, Nilo Peçanha st., Petrópolis, Natal, RN 59012-300, Brazil; E-Mails: irami.filho@uol.com.br (I.A.F.); aldo@ufrnet.br (A.C.M.); 4 Laboratory of Pathology, Department of Pathology, Federal University of Rio Grande do Norte (UFRN), General Gustavo Cordeiro de Farias Ave., Petrópolis, Natal, RN 59012-570, Brazil; E-Mails: keyla.rocha@uol.com.br (K.B.F.R.); babethmaia@hotmail.com (E.M.O.); 5 University Hospital Onofre Lopes, Department of Integrated Medicine, Federal University of Rio Grande do Norte (UFRN), 620, Nilo Peçanha st., Petrópolis, Natal, RN 59012-300, Brazil; E-Mail: eduardofaria@ufrnet.br; 6 Department of Biochemistry and Molecular Biology, Federal University of Paraná (UFPR), Cento Politécnico S/N, Jardim das Américas, Curitiba, PR 81531-990, Brazil; E-Mail: sassaki@ufpr.br

**Keywords:** water-insoluble glucan, polysaccharide, yeast, immunomodulator, tissue repair

## Abstract

Water-insoluble glucan was isolated from the baker’s yeast *Saccharomyces cerevisiae*. The yeast cells were treated with alkali and the residue then with acid. Chemical and NMR (1D and 2D) analyses showed that a linear (1→3)-β-glucan was purified that was not contaminated with other carbohydrates, proteins or phenolic compounds. The effects of the glucan on wound healing were assessed in human venous ulcers by histopathological analysis after 30 days of topical treatment. (1→3)-β-glucan enhanced ulcer healing and increased epithelial hyperplasia, as well as increased inflammatory cells, angiogenesis and fibroblast proliferation. In one patient who had an ulcer that would not heal for over 15 years, glucan treatment caused a 67.8% decrease in the area of the ulcer. This is the first study to investigate the effects of (1→3)-β-glucan on venous ulcer healing in humans; our findings suggest that this glucan is a potential natural biological response modifier in wound healing.

## 1. Introduction

Wound healing is an extremely complex biological process that is regulated by molecular and cellular events to promote tissue repair. Wound healing requires the proliferation, differentiation and recruitment of numerous cell types, including keratinocytes, endothelial cells, fibroblasts and immune cells, such as neutrophils, monocytes/macrophages, lymphocytes and dendritic cells [1].

After an injury, a platelet plug is formed that is surrounded by a fibrin matrix, which becomes a scaffold for infiltrating cells to adhere. During the early stage, recruited neutrophils phagocytose and degrade the devitalized tissue and release pro-inflammatory cytokines, such as interleukin-1β (IL-1β), interleukin-6 (IL-6) and tumor necrosis factor-α (TNF-α). These cytokines are essential for the activation of keratinocytes, fibroblasts and additional cells [2,3]. Subsequently, monocytes migrate to the wound and differentiate into macrophages. The macrophages act similarly to the neutrophils and produce pro-inflammatory cytokines, as well as reactive oxygen and nitrogen intermediates, which play an important role in host defense. In addition, macrophages release growth factors, including platelet-derived growth factor (PDGF), transforming growth factor (TGF)-β, TGF-α, basic fibroblast growth factor (bFGF) and vascular endothelial growth factor (VEGF). These factors stimulate angiogenesis, stimulate extracellular matrix synthesis by local fibroblasts and promote granulation tissue formation and reepithelialization [4–6].

During wound healing, T-lymphocytes produce growth factors and function as immunological effector cells, which play a role in the activation of macrophages and B-lymphocytes. T-lymphocytes also function as regulatory cells and control the proliferation phase of wound healing. B-lymphocytes have several essential functions that regulate the immune response, including cytokine, growth factors and immunoglobulin production, as well as antigen presentation and regulation of T-lymphocyte activation and differentiation [7,8]. Nevertheless, the healing process can occur very slowly in some instances and the wound has an increased risk of becoming contaminated and infected. Thus, several research groups have sought to identify molecules from natural sources that can accelerate wound healing, such as glucans.

Glucans are glucose polymers with an α- or β-type glycosidic chain. Glucans are present in fungi, plants, algae and bacteria. In fungi, β-glucans are the major components of the cell wall and are usually linked to proteins, lipids and other polysaccharides, such as mannan [9]. One major source of β-glucan is the baker’s yeast *Saccharomyces cerevisiae*. In this yeast, β-glucans primarily exist in the (1→3)-β-linked backbone form with (1→6)-β-branches [10]. However, a minor amount of (1→3)-β-glucan also exists [11].

The β-glucans are recognized as pathogen-associated molecular patterns (PAMPs) by several mammalian immune cell receptors, such as dectin-1, toll-like receptors, complement receptor 3 (CR3) and lactosylceramide. These receptors allow the β-glucan to interact with immune cells, such as neutrophils, macrophages and lymphocytes. These interactions activate several intracellular pathways that are responsible for the immunopharmacological properties of β-glucan; however, the complete mechanism β-glucan function is not yet fully understood [12].

In addition to interacting with immune cells, β-glucan also interacts with fibroblasts. Wei *et al.* [13] reported that (1→3)-β-glucan directly stimulated collagen biosynthesis in normal human dermal fibroblasts by activating two families of transcription factors, activator protein 1 (AP-1) and specific protein 1 (SP-1).

This polysaccharide has also been reported to increase wound healing in animals subjected to skin incision, colon anastomosis and burns. Depending on the route of administration, β-glucan enhanced wound healing by increasing macrophage infiltration and collagen deposition, by stimulating tissue granulation and by promoting reepithelialization [14–16]. Only one human wound healing study has been performed [17]. In this study, the authors demonstrated that (1→3),(1→6)-β-glucan was effective as a topical treatment for partial thickness burns in pediatric patients. However, there are no reports on the application of (1→3)-β-glucan in human wound healing.

Venous ulcers are cutaneous wounds that result from chronic venous insufficiency; they are responsible for approximately 80%–85% of all ulcers that occur on the lower limbs. Venous ulcers represent a serious public health problem throughout the world. These wounds are difficult to heal because of their recurrence, high risk of infection and the high cost of treatment. These ulcers cause significant morbidity, pain, work productivity loss and decrease the quality of life in affected patients [18].

Based on these considerations, the purpose of this study was to purify water-insoluble (1→3)-β-glucan, assess its chemical structure and evaluate its effect on venous ulcer healing in humans.

## 2. Results and Discussion

### 2.1. Structural Characterization

Chemical analysis indicated that the extracted polysaccharide was composed solely of glucose. Proteins and phenolic compounds were not detected in the sample, which indicates that we successfully purified a homoglucan from *S. cerevisiae*.

**Figure 1:**
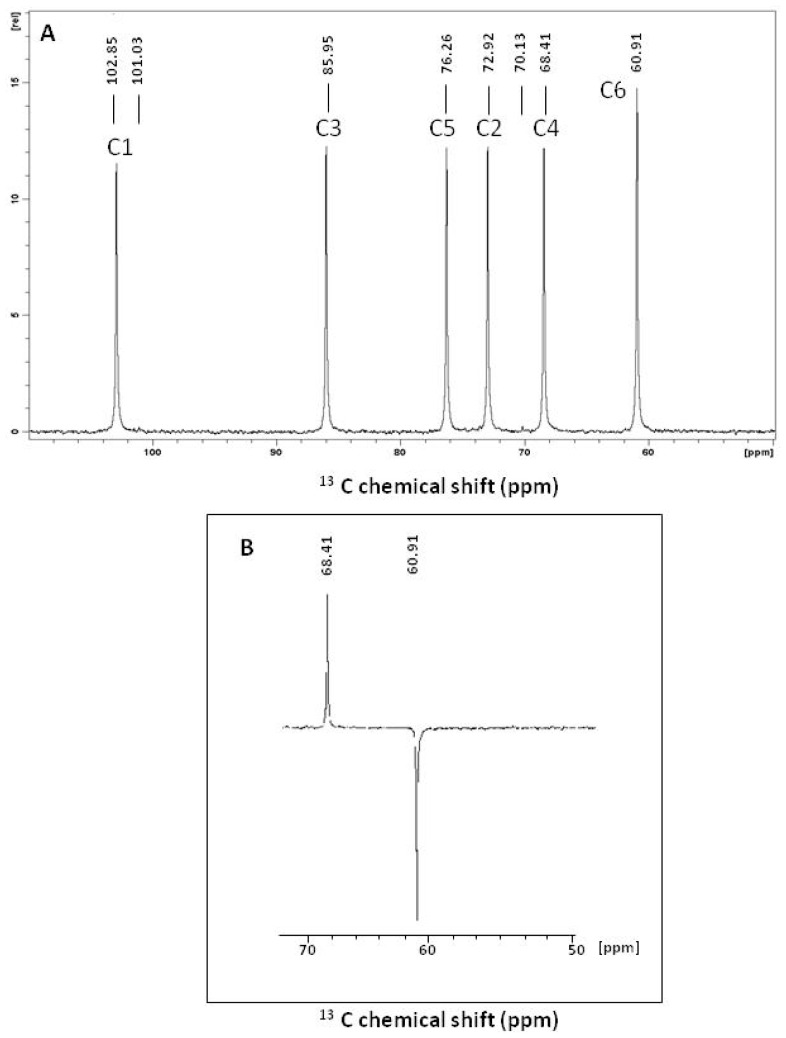
^13^C NMR spectrum of the water-insoluble glucan isolated from the baker’s yeast *Saccharomyces cerevisiae*. (**A**) The number of carbon atoms was labeled on each peak to indicate the position. (**B**) The inset shows a DEPT-135 analysis of the C-6 region.

The ^13^C NMR spectrum of the water-insoluble glucan showed six carbon signals at ∂ 102.86 (C-1) ppm, which correspond to β anomeric carbons, as well as at ∂ 85.96 (C-3), 76.27 (C-5), 72.94 (C-2), 68.41 (C-4) and 60.91 (C-6) ppm (Figure 1A). This indicates that the polysaccharide contains homogeneous repeat monosaccharide units. Furthermore, the ^13^C NMR spectrum revealed no evidence of α anomeric configuration. Confirmation of free carbon 6 (C6) at ∂ 60.91 ppm was established by the inverted CH2 signal in DEPT-135 ^13^C NMR (Figure 1B).

**Figure 2:**
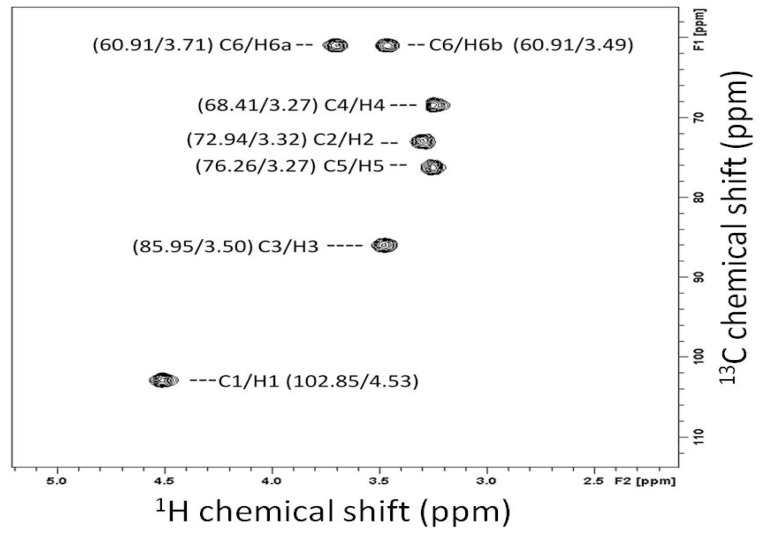
^1^H and ^13^C HSQC spectra of the water-insoluble glucan isolated from the baker’s yeast *Saccharomyces cerevisiae*. The ^13^C NMR and ^1^H NMR spectra are displayed on the vertical and horizontal axes, respectively.

All of the protons were assigned to carbons using the two-dimensional (2D) ^1^H-^13^C NMR heteronuclear single quantum correlation (HSQC) spectrum (Table 1 and Figure 2). All of the NMR chemical shifts are comparable to literature values [19–23]. The results from the ^1^H-^13^C NMR spectrum confirmed that the water-insoluble compound is a (1→3)-β-linked glucan that is similar to laminarin, which is a standard (1→3)-β-glucan [24].

Linear (1→3)-β-glucans are present in several microorganisms, such as in the capsular polysaccharides of gram-negative bacteria that belong to the rhizobiaceae (e.g., the *Agrobacterium* sp.) [25] the gram-positive bacteria *Cellulomonas flavigena* [26] and the sclerotia of the basidiomycete fungus *Poria cocos* [27]. In *S. cerevisiae*, (1→3),(1→6)-β-glucan, which comprises approximately 50% of the cell wall, forms a core where the non-reducing termini are covalently linked either to chitin, (1→6)-β-glucan or mannoprotein, which together compose approximately 40% of the wall. Minor amounts of (1→3)-β-glucan are additionally present in *S. cerevisiae* [11].

In this work, we isolated (1→3)-β-glucan from *S. cerevisiae*. The purified glucan was not contaminated with other carbohydrates, proteins or phenolic compounds, which has been historically difficult to achieve. Because there have been few studies that have assessed the biological activity of this glucan, and because there are no reports that have assessed the effect of (1→3)-β-glucan on venous ulcer healing in animals or humans, we sought to purify the compound and assess its effect on human ulcer wound healing.

### 2.2. Study Subjects

We followed 12 patients who had venous ulcers; within this study group, 1 patient had 2 ulcers. Nine patients (75%) were women and three (25%) were men. The patient age ranged from 42 to 75, with an average of 59 +/− 10.5 years.

Topical immunotherapy was applied to 13 venous ulcers, of which 9 (69.2%) were located in the middle third of the leg. The average ulcer lifespan was 142.9 months (range of 8 to 264 months) and the majority of the ulcers (61.5%) were recurrent wounds.

In all of the patients, the ulcers were associated with chronic venous insufficiency. The patients had careers that favored venous stasis where they remained seated or standing for prolonged time periods. In addition, 6 (50%) patients had hypertension, of whom 3 (25%) also had diabetes *mellitus*. These factors probably cooperated in the development of nonhealing venous ulcers.

### 2.3. Tissue Sample Collection and Histopathological Analysis

For the qualitative histopathological analysis, 13 ulcer biopsy fragments were evaluated before therapy initiation (day 0) and 13 fragments were evaluated on day 30 of glucan treatment, for a total of 26 fragments. In addition, the number of lymphocytes, plasmocytes and neutrophils were determinate.

Epithelial hyperplasia was present at the ulcer edges in all of the specimens at day 0. Five specimens (38.5%) had reactive and reparative epithelial changes in the stratified squamous epithelium that were associated with hyperplasia. Keratinocyte reactivity was clearly demonstrated by the presence of anisokaryosis, anisonucleose and other changes. Inflammation was apparent in all of the specimens and inflammatory cells: lymphocytes (mean: 25.62 cells/field), plasmocytes (mean: 25.23 cells/field) and neutrophils (mean: 43.31 cells/field) were present.

**Figure 3:**
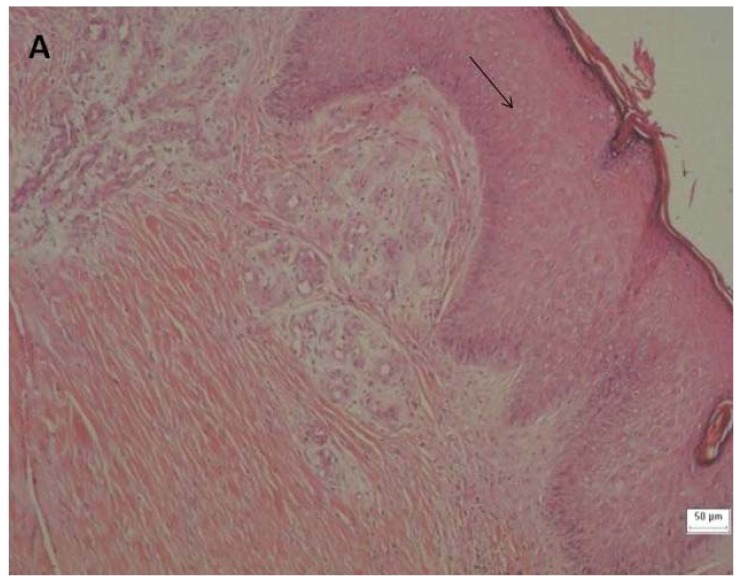

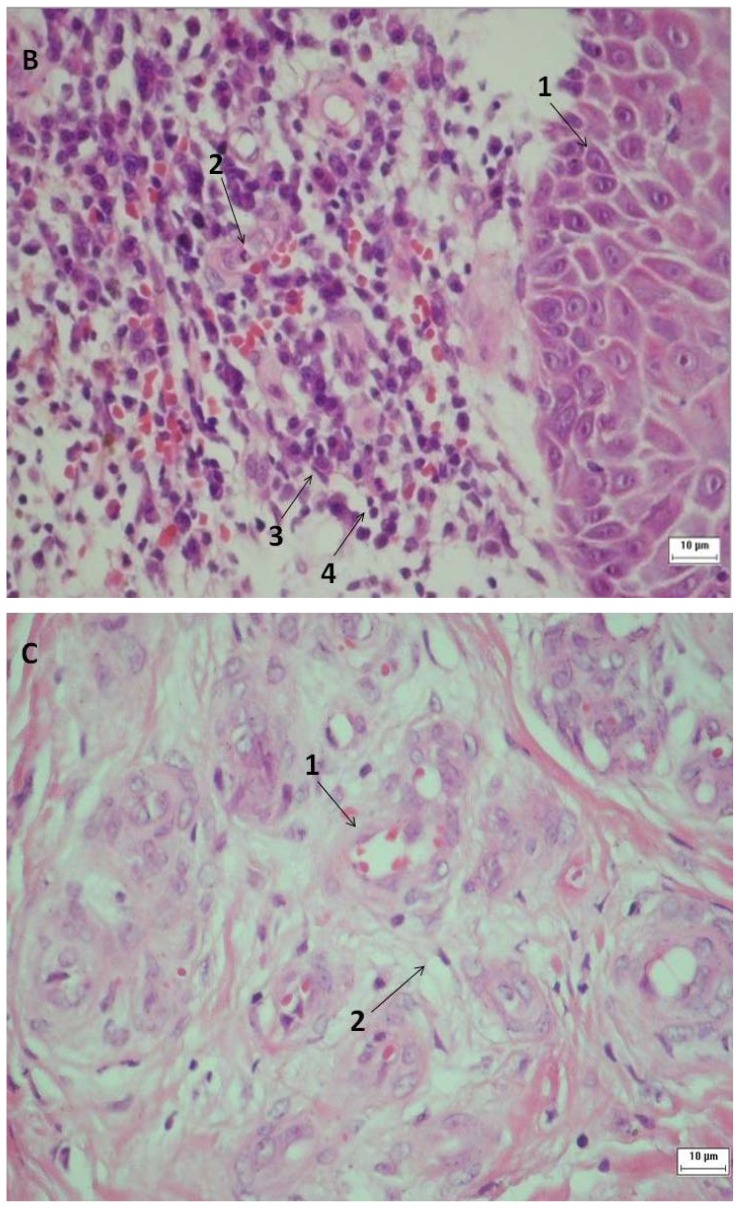
Histology of the venous ulcers. The tissue was stained with hematoxylin and eosin (H & E) to visualize the cellular morphology. Samples A, B and C were collected prior to (1→3)-β-glucan treatment. The following magnifications are shown: 100 × H & E stain for A; 400 × H & E stain for B and C. (**A**) The arrow indicates epithelial hyperplasia at the edge of the ulcer. (**B**) Arrow No. 1 shows reactive and reparative epithelial changes in the stratified squamous epithelium and the other arrows show inflammatory cell infiltration, including neutrophil (arrow No. 2), plasmocyte (arrow No. 3) and lymphocyte (arrow No. 4). (**C**) Arrow No. 1 indicates angiogenesis associated with edema and arrow No. 2 indicates fibroblast.

**Figure 4:**
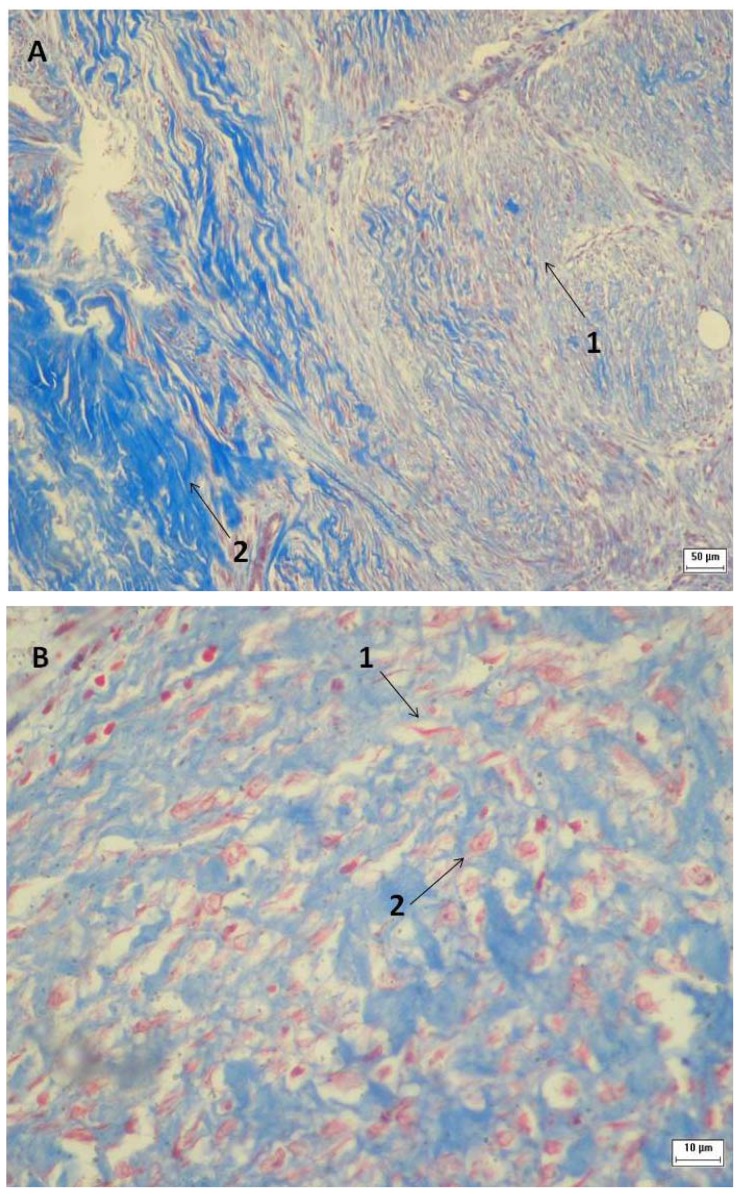

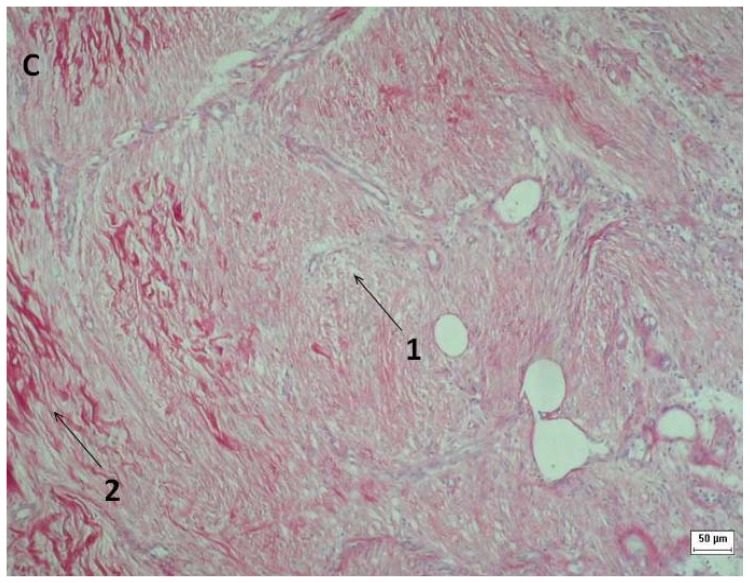
Venous ulcer histology. The tissues in samples A and B were stained with Masson’s trichrome, while sample C was stained with picrosirius red. Samples A, B and C were obtained prior to (1→3)-β-glucan treatment. The magnifications shown are as follows: 100 × for samples A and C; 400 × for sample B. (**A** and **C**) Arrow No. 1 indicates new collagen deposition in the ulcerated area and arrow No. 2 indicates senescent collagen deposition deep within then venous ulcer. (**B**) Arrow No. 1 indicates senescent fibroblast and arrow No. 2 indicates young fibroblast amid collagen deposition in the ulcerated area.

Angiogenesis was present in 100% of the specimens at day 0. Well-formed vessels with a well-defined thickness and edema were observed. Fibroblast proliferation and collagen fibrosis were detected in all of the specimens. Senescent fibroblasts were present in the ulcer margin and there was a predominance of new fibroblasts in the ulcerated area. The collagen fibers on the ulcer edges were well formed and had a homogeneous distribution pattern. The collagen fibers in the ulcerated area were delicate and were distributed in the upper dermis. For the remaining two-thirds of dermis, there was a dense distribution of fibers that was associated with inflammation and edema (Figures 3 and 4).

New biopsies were taken after 30 days of glucan treatment. By day 30, histopathology indicated that there was an increase in epithelial hyperplasia. Additionally, 12 specimens (92.3%) had reactive and reparative epithelial changes of the stratified squamous epithelium that were associated with hyperplasia, of which 2 (15.4%) specimens had reepithelialization zones that covered the previously denuded areas. These data suggest that an increase in epithelial cell migration occurred.

Inflammation was present in all of the samples and was identified an increase in the number of inflammatory cells: lymphocytes (mean: 31.23 cells/field), plasmocytes: (mean: 42.85 cells/field) and neutrophils: (44.54 cells/field). The differences between the number of inflammatory cells before therapy initiation (day 0) and after 30 days of glucan treatment (day 30) were evaluated and the increase in the number of plasmocytes was statistically significant (*p* = 0.018). The number of inflammatory cells may have been influenced by the ability of the glucan to promote cell proliferation in local ulcerated regions and subsequential leukocyte recruitment. As previously described, leukocytes are essential for the production of growth factors, cytokines and other inflammatory mediators that play a key role in wound healing and host defense.

Angiogenesis was present in 100% of the specimens and maintained the same pattern before and after glucan treatment. Fibroblast proliferation was observed in 100% of the specimens and the number of fibroblasts was higher on day 30 compared to day 0. Additionally, there were senescent fibroblasts in the sample margin and an increased number of new fibroblasts in the ulcerated area. Collagen fibrosis was present in the same pattern at day 30 compared to day 0 (Figures 3 and 4). Fibroblasts contribute to granulation tissue formation by synthesizing collagen, elastin, fibronectin, glycosaminoglycan and proteases (components of extracellular matrix). They also produce cytokines that promote keratinocyte proliferation and migration and promote myofibroblast differentiation to promote wound closure [1].

The current standard of treatment for chronic venous ulcers is the application of compression bandages to reduce venous pressure and edema as well as to improve venous return [28,29]. However, venous ulcers do not always heal in response to the standard therapies, especially ulcers that have persisted for long periods of time or reoccur.

Experimental studies with other wounds types have reported that topical or systemic β-glucan administration enhances wound healing. Leibovich and Danon (1980) [30] reported that there was a higher number of macrophages in the early inflammatory stage of repair. In mice, reepithelialization and the onset of fibroplasias commenced at an earlier stage when (1→3)-β-glucan was topically applied to the wound compared with the control group. Topical application of (1→3)-β-glucan to wounds in mice, rats and guinea pigs accelerated reepithelialization and increased fibroblast proliferation and fibrogenesis by activating macrophages [31]. In another study, (1→3)-β-glucan was administered intravenously and topically in rats that had dorsal skin incisions; treatment enhanced macrophage function and increased the early wound strength, which may have been strengthened by increased collagen cross-linking [32].

Portera *et al.* [33] reported that macrophage modulation via intravenous administration of (1→3)-β-glucan phosphate increased tensile strength in experimental colon anastomosis and skin incisions in rodents. Additionally, they observed a positive correlation between phosphate (1→3)-β-glucan treatment, wound tensile strength and collagen biosynthesis.

Artificial skin, which was obtained from cultured fibroblasts and keratinocytes in a medium containing (1→3),(1→6)-β-glucan and gelatin, was applied to experimentally induced wounds in athymic mice and promoted reepithelialization [15].

Toklu *et al.* [16] reported that local and oral administration of (1→3),(1→6)-β-glucan protected against burn-induced oxidative tissue damage in rats. Topical application of aminated (1→3)-β-glucan (AG) improved wound healing in mice with diabetes mellitus and histologic examinations of the wounds revealed vascularized granulation tissue that was rich in cells and had increased reepithelialization compared with the placebo group [14]. Delatte *et al.* [17] reported that (1→3),(1→6)-β-glucan associated with collagen matrix was an effective treatment that significantly decreased post-injury pain in partial thickness burns in pediatric patients.

### 2.4. Ulcer Area Imaging and Measurement

**Figure 5:**
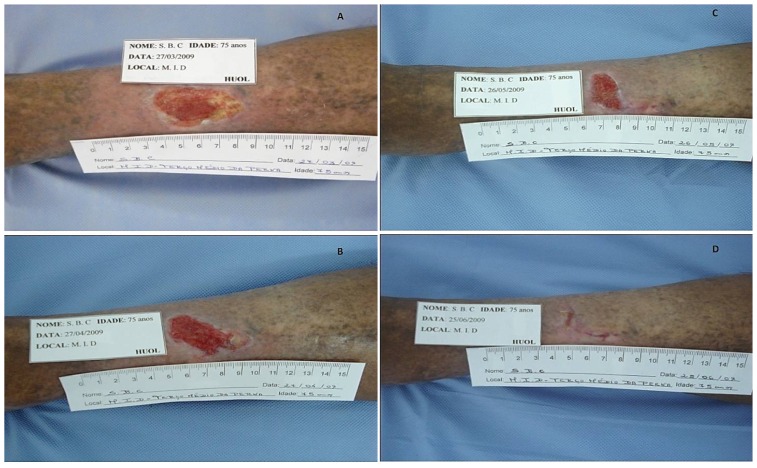

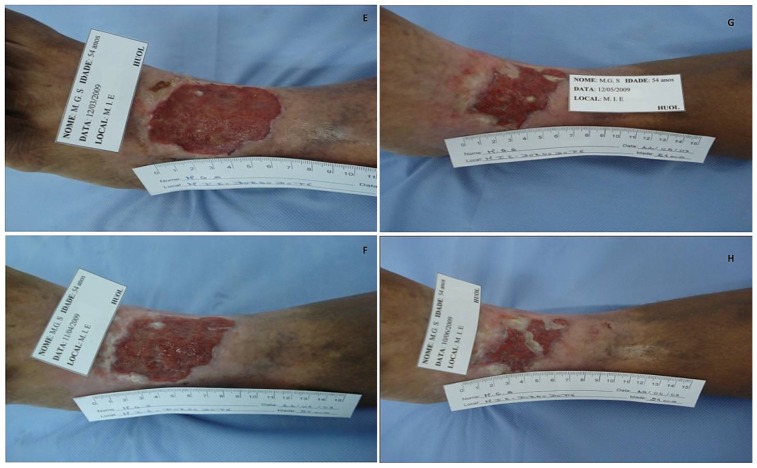
Ulcer healing over time with (1→3)-β-glucan treatment. The images represent the progress of ulcer healing over the treatment time course for 2 patients. (**A**) A venous ulcer in the right leg at day 0 that measures 8.38 cm^2^. (**B**) The venous ulcer shown in (**A)** at day 30, which measures 4.84 cm^2^. (**C**) The venous ulcer shown in (**A**) at day 60, which measures 2.92 cm^2^. (**D**) The venous ulcer shown in (**A**) at day 90, which has completely healed. (**E**) A venous ulcer on the left foot at day 0 that measures 27.3 cm^2^. (**F**) The venous ulcer shown in (**E**) at day 30, which measures 25.62 cm^2^. (**F**) The venous ulcer shown in (**E**) at day 60, which measures 15.87 cm^2^. (**H**) The venous ulcer shown in (**E**) at day 90, which measures 8.78 cm^2^.

Initially, the venous ulcers possessed an average area of 27.51 cm^2^ (range of 8.38 to 66.89 cm^2^). Although these wounds were difficult to heal because of their duration, recurrence and area, the average of the reduction percentage on ulcer area was 11.3% after 30 days of glucan treatment. By day 90, one venous ulcer had healed completely, and 7 ulcers exhibited an average area of 16.48 cm^2^ (range of 2.08 to 41.42 cm^2^); the average of the reduction percentage on ulcer area was 55.23% at day 90. Figure 5A–D shows a venous ulcer on a right leg that healed completely after 90 days of glucan treatment. Interestingly, the ulcer shown in Figure 5E–H, which has not healed over the past 15 years, reduced in size by 67.8% after only 3 months of glucan treatment.

## 3. Experimental Section

### 3.1. Materials

Sodium hydroxide (NaOH), hydrochloric acid (HCl), 99.8% ethyl alcohol (EtOH), hematoxylin and eosin yellow were purchased from Vetec^®^ (Rio de Janeiro, Brazil). *S. cerevisiae* (baker’s yeast) was purchased from Fleischmann^®^ (Rio de Janeiro, Brazil). All the other reagents used in this work were analytical grade.

### 3.2. Isolation of Water-Insoluble (1→3)-β-Glucan

Water-insoluble (1→3)-β-glucan was isolated from *S. cerevisiae.* The yeast cells were treated with alkali and the residue then with acid at the Laboratory of Clinical Immunology, UFRN according to the method described by Hassid *et al.* [34] with some modifications. Briefly, 1.5 kg of dry yeast was digested in 3% NaOH (2 L) at 80 °C for 6 hours and then incubated at room temperature overnight. The supernatant was discarded and the extraction was repeated. The residue was subsequently acidified with approximately 2.8 L of 4 M HCl and digested for several hours at 80 °C. After the digested mixture was incubated overnight at room temperature, the supernatant was discarded and the residue was subjected to a new digestion with 3% HCl (2 L). The overall yield was approximately 40 g.

### 3.3. Structural Characterization

The total sugar concentration was estimated with a phenol-H_2_SO_4_ reaction [35] with D-glucose as the standard. The total protein concentration was measured with the Bradford method [36] using bovine serum albumin (BSA) as the standard. The total phenolic compound concentration was determined by the Folin-Ciocalteu colorimetric method using gallic acid as the standard [37]. The polymers were hydrolyzed (5 M TCA, 100 °C, 2 h) and the sugar composition was determined by high performance liquid chromatography (HPLC) (Merck Hitachi Elite LaChrom^®^) on a machine equipped with a refractive index detector and a LichroCART^®^ 250-4 column. Arabinose, galactose, glucose, fucose, mannose, rhamnose and xylose were used for references.

The β-glucan (50 mg) was dissolved in 800 μL of DMSO-d_6_. Nuclear magnetic resonance (NMR) spectra (^13^C and DEPT-135) were obtained with a 400 MHz Bruker Avance III spectrometer in an inverse 5 mm gradient probe head at 70 °C. ^1^H-^13^C NMR heteronuclear single quantum correlation (HSQC) spectra were recorded using states-times proportion phase incrementation for quadrature detection in the indirect dimension. The HSQC spectra were performed with 1024 × 256 points and globally optimized and alternating-phase rectangular pulses for decoupling were applied. The chemical shifts are expressed in δ relative to acetone at δ 32.77 (^13^C) and 2.21 (^1^H), based on sodium 2,2-dimethyl-2-silapentane-3,3,4,4,5,5-*d*6-5-sulfonate (DSS) at δ = 0.00 for ^13^C and ^1^H in accordance with IUPAC recommendations.

### 3.4. Study Subjects

A nonrandomized clinical trial with intragroup comparisons over time was performed. The study was approved by the Research Ethics Committee of the University Hospital Onofre Lopes, HUOL/UFRN, Brazil (protocol number: 147/07). The study included male and female venous ulcer patients who were older than 18. The patients were admitted to the Vascular Surgery Ambulatory at the University Hospital Onofre Lopes, HUOL/UFRN. The exclusion criteria were the following: autoimmune disease, heart, renal or liver insufficiency and/or malignancies. Data regarding the ulcer location, duration, status (new or recurrent) and surface area were collected. The baseline patient medical history information included the following: diabetes mellitus, hypertension, endocrine disease, peripheral vascular disease, connective tissue disease, musculoskeletal disease and neurological disorders.

### 3.5. (1→3)-β-Glucan Treatment

Powdered (1→3)-β-glucan was autoclaved and dispersed in cream with crodabase CR2 to a final concentration of 3%; the cream was applied directly to the ulcer bed after the area was cleaned with a 0.9% sterile saline solution. The ulcers were subsequently covered with nonadherent gauze, moistened with 0.9% sterile saline and covered with a crepe bandage. This procedure was performed daily for up to 90 days.

### 3.6. Tissue Sample Collection and Histopathological Analysis

A venous ulcer biopsy was taken from all of the patients before therapy initiation (day 0) and after 30 days of glucan treatment (day 30). Prior to tissue sample collection, 2% xylocaine was injected for local anesthesia. The biopsy was extracted with a n° 15 blade scalpel and included the ulcerated area and the ulcer edge. The tissue was fixed in 10% formalin for 24 hours, processed according to the Laboratory of Pathology/UFRN protocol and stained with hematoxylin and eosin to visualize the skin structure, as well as the cellular and tissue components. The tissue was also stained with Masson’s trichrome and picrosirius red to identify the collagen fibers. The following parameters were assessed for the histopathological analyses: epithelial hyperplasia, inflammatory response, angiogenesis, fibroblast proliferation and collagen fibrosis. Histomorphometric analysis of inflammatory cells (neutrophils, lymphocytes and plasmocytes) was performed and 7 microscopic high power fields per specimen were counted (magnification 400×). The number of inflammatory cells in each field was counted and the mean of all fields was calculated. The differences between the number of inflammatory cells before therapy initiation (day 0) and after 30 days of glucan treatment (day 30) were evaluated by the Wilcoxon test. *p* < 0.05 was considered statistically significant.

### 3.7. Ulcer Imaging and Quantification of the Ulcer Area

To monitor the healing progress, color images were acquired and the ulcer area was quantified with a computer program (AutoCAD 2008^®^) from a traced outline of the venous ulcer on transparent film. These procedures were performed before the initiation of therapy (day 0) and once every week for up to 90 days.

## 4. Conclusions

We purified a linear (1→3)-β-glucan from *Saccharomyces cerevisiae* using the method described by Hassid *et al.* [34]. (1→3)-β-glucan enhanced venous ulcer healing and increased epithelial hyperplasia, as well as increased the number of plasmocytes and fibroblast proliferation. This is the first study that investigated the effect of (1→3)-β-glucan in venous ulcer healing in humans; our findings suggest that this glucan is a potential natural biological response modifier for wound healing. Further studies are needed to assess the therapeutic benefits of this compound and to elucidate with more details the mechanisms responsible for wound healing.

## Figures and Tables

**Table 1:** The assignments of ^13^C NMR and HSQC spectrum.

Sugar Residue	^13^C/^1^H (ppm)							Ref.

	1	2	3	4	5	6		

						6a	6b	
→3)-β-Glc-(1→	102.85	72.94	85.95	68.41	76.26	60.91	3.49	a
	4.53	3.32	3.50	3.27	3.27	3.71		
→3)-β-Glc-(1→	102.49	72.46	85.76	68.18	76.02	60.67	3.51	b
	4.55	3.34	3.51	3.31	3.31	3.74		
→6)-β-Glc-(1→	104.66	74.71	76.57	71.14	77.25	70.48	3.75	c
	4.42	3.22	3.40	3.35	3.53	4.12		
→3,6)-β-Glc-(1→	102.96	73.04	85.76	68.53	74.96	68.68	3.58	d
	4.54	3.34	3.54	3.26	3.52	4.08		
→3,4)-β-Glc-(1→	103.02	73.69	85.34	68.99	76.48	61.97		e
	-	-	-	-	-	-		

a: This work;

b: Freimund *et al.* [21];

c: Bi *et al.* [19];

d: Tada *et al.* [23];

e: Roubroeks *et al.* [22].
